# The Practice of Online Medical Care at Juntendo Hospital in Response to the Coronavirus Pandemic

**DOI:** 10.14789/jmj.JMJ23-0006-R

**Published:** 2023-05-20

**Authors:** RYOHEI KUWATSURU

**Affiliations:** 1 Department of Radiology, Graduate School of Medicine, Juntendo University, Tokyo, Japan; 1 Department of Radiology, Graduate School of Medicine, Juntendo University, Tokyo, Japan

**Keywords:** coronavirus pandemic, remote medical care, online medical consultation recommendation, preclinical consultation

## Abstract

The numbers of coronavirus (COVID-19) infections have exploded in Japan since mid-March 2020, making it difficult for outpatients to visit our hospital (Juntendo Hospital in Tokyo). For this reason, the hospital expanded the use of online medical care in May 2020 to ensure uninterrupted medication treatment for outpatients who could not attend in person. Although the number of outpatient visits in person was reduced, patients were still able to consult our clinic and receive their medication through online medical care via audio–video systems. This paper discusses the background to this situation, as well as the guidelines, the medical fee system, and the advantages and disadvantages of online medical care in Japan.

The use of online medical care has widely expanded nationwide in response to the coronavirus pandemic. Juntendo Hospital is a general hospital affiliated with a private university and located near Tokyo Station, which is convenient not only for patients in the neighborhood but also for those in rural areas. The hospital has 1,051 beds and approximately 3,700 outpatients per day. Our hospital had been providing online medical care prior to the pandemic, but the number of patients was limited, and the recent pandemic prompted the hospital to begin a full-scale operation of online medical care. This paper reviews the implementation of online medical care at our hospital, with reference to the various laws, guidelines, and medical fee systems surrounding online medical care in Japan.

## Guidelines for the appropriate implementation of online medical treatment

### Environment surrounding online medical care

Information and telecommunication devices have made great advances in recent years, and their use has spread rapidly in Japan. The relationship between medical care through the use of these devices and Article 20 of the *Medical Practitioners Act* (Act No. 201 of 1948), which prohibits medical treatment without examination, was clarified in a 1997 notice, “Medical examinations applying ICT [Information and Communications Technology],” issued by the Ministry of Health, Labour and Welfare (MHLW). This has made it possible to provide medical care by using modern telecommunications devices (e.g., online medical care).

In addition, from the perspective of information security and other issues when medical information is handled electronically, *Security Guidelines for Medical Information Systems* were published by the MHLW in 2005 and have been revised several times. The use of telecommunications devices in medical practice can reform the way in which physicians work and help to overcome the uneven distribution of physicians in Japan. *Guidelines for the Appropriate Implementation of Online Medical Treatment* were established by the MHLW in March 2018. They were partly revised in January 2022 to clarify minimum requirements, recommendations, and concepts, and to promote the implementation of online medical care with which physicians, patients, and related parties can feel comfortable in terms of safety, necessity, and effectiveness.

In a further interpretation of the Medical Practitioners Act, the “Medical examinations applying ICT” notice was revised in 2003 and 2011. Article 1-2 of the *Medical Care Act* (Act No. 205 of 1948) stipulates that medical care must be provided by hospitals, clinics, long-term healthcare facilities, dispensing pharmacies, and other facilities that provide medical care (hereinafter referred to as “medical institutions”) and in the homes of medical care recipients (meaning a home or “other place” as specified by an Order of the MHLW). The Enforcement Regulations on the *Medical Care Act* (Order of the Ministry of Health and Welfare No. 50 of 1948) stipulate that the “other places” prescribed by Order of the MHLW, as provided in Article 1-2, paragraph 2, of the Act, are nursing homes for the elderly, intensive care homes for the elderly, low-cost homes for the elderly, fee-based homes for the elderly, and places where medical care recipients can live with medical treatment.

## Definitions of terms used in the guidelines for the appropriate implementation of online medical treatment, and scope of the guidelines

This section defines the terms related to online medical care.

### Remote medical care

Remote medical care is an umbrella term that refers to health-promoting medical care via telecommunications devices. This includes telehealth medical consultations that can be performed by non-doctor, such as giving general information on common diseases and symptoms.

### Online medical care

As a part of remote medical care, online medical care refers to the act of examining and diagnosing patients and communicating the results of diagnoses, and prescriptions, in real time from physician to patient via telecommunications devices.

### Online medical consultation recommendation

An online medical consultation recommendation is a form of remote medical care in which a physician examines a patient via telecommunications devices and recommends in real time that the patient visit a medical institution. It involves the minimum medical judgment appropriate for the individual patient's physical and mental condition. This includes determining and recording the name of the suspected disease on the basis of the patient's signs and symptoms; collecting information on the patient's physical and mental condition, such as by interview; and selecting the appropriate department to be consulted. Follow-up and non-clinical recommendations, including home treatment with over-the-counter drugs, can also be implemented. With such a recommendation, the patient is not informed of their diagnosis or prescribed drugs.

### Preclinical consultation

A preclinical consultation is an act of confirming a patient's symptoms and medical information via real-time exchange between the physician and patient via an audio–video system. This may occur when a physician other than the physician who already has a direct relationship with the patient (e.g., a family doctor through regular face-to-face visits) intends to practice online medical care from the first online consultation onward (except in cases where the physician already has enough medical information on the patient).

Online medical care will be followed when appropriate information can be obtained and both the physician and the patient mutually agree that such care is feasible.

### Coverage of online medical care

**Figure 1:**
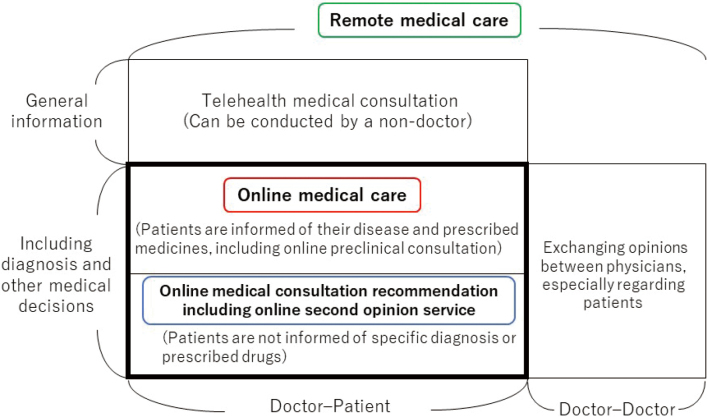
Relationship between online medical care, online medical consultation recommendation, and remote medical care

Figure 1 illustrates the relationships among remote medical care, online medical care, online medical consultation recommendation, and telehealth medical consultation. The *Guidelines for the Appropriate Implementation of Online Medical Treatment* cover the online medical care and online medical consultation recommendation shown in this figure. They do not include remote medical care in the form of telehealth medical consultation. The doctor-doctor relationship in the figure refers to the exchange of opinions between physicians, especially regarding patients, which is not included in online medical care.

## Online medical care at our hospital

This section describes the practice of online medical care at our hospital.

### Steps to take before starting online medical care

Ambulatory care physicians first take e-learning courses on online medical care. Thereafter, they practice online medical care while referring to the *Guide to Online Clinical Practice in Primary Care*^1)^ (v. 1.0, released on 20 May 2020). Online medical care is a medical practice that uses telecommunications devices with video communication functions and is quite different from telephone medical care.

Physicians next refer to *Guidance for Primary Care Initial Clinical Practice in Clinics and Hospitals for Coronavirus* (*COVID-19*) *Infections*^2)^ (v. 3.0 released on 7 November 2020; v. 2.0 released on 30 April 2020). There are two types of online medical care: “online medical care in normal times” and “online medical care as a provisional measure”. The provisional measure, declared by MHLW as an emergency response to the rapid spread of the coronavirus, started in April 2020 and lasted until March 31, 2022. In accordance with the MHLW policy, our hospital also started implementing the provisional measure in April 2020 and expanded its use from May 2020. During this period, online medical care was provided, and medical fees were calculated on the basis of the provisional measure.

Five conditions must be considered to determine whether online medical care is appropriate in outpatient settings:^1)^ (1) whether or not a doctor–patient relationship has been established; (2) whether or not an online medical care provider is available; (3) whether or not the patient is registered with a medical institution; (4) whether it is a first visit or a follow-up visit in terms of medical fees; and (5) whether the patient's symptoms are acute or chronic, and mild or severe. The primary care physician should refer to the *Guide to Online Clinical Practice in Primary Care* to determine which cases are easy to treat appropriately online and which cases should be carefully considered and therefore should be treated in person.

### Implementing online medical care

**Figure 2:**
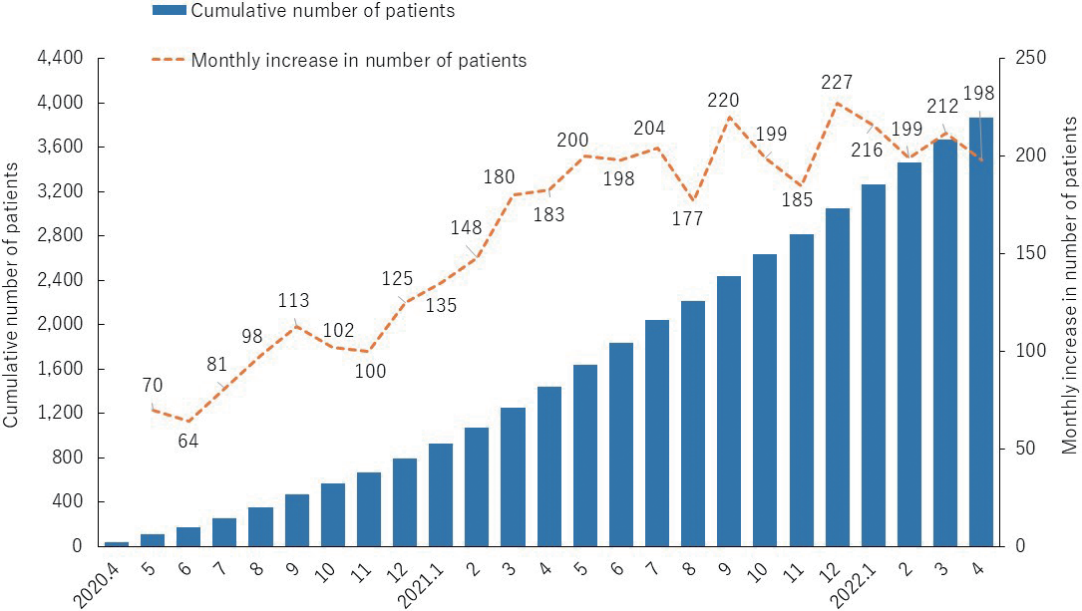
Numbers of online medical care patients at our hospital from April 2020 to April 2022.The bar and line graphs show the cumulative number of patients and the number of patients for each month, respectively.

The ambulatory care physician first determines whether the patient is eligible for online medical care. The physician then contacts the hospital's Online Medical Care Support Department to coordinate the examination. The physician and the Support Department set the day and time of the online medical examination. Specifically, the Support Department contacts the patient before the day of the examination to set up the examination. We have implemented online medical care via Zoom (Zoom Video Communications Japan, https://explore.zoom.us/ja/about/). The advantages of Zoom are: (1) it is free of charge within 40 minutes whereas using an existing online medical care system would cost a lot of money; (2) Easy to set up; (3) Easy to operate; (4) Security updates are frequent. On the contrary, the drawback of Zoom is that if the meeting ID and password are leaked to outsiders, others can access the meeting. Considering the above, the following security measures are taken when using Zoom: (1) Always use the latest version of the software by updating; (2) Zoom connection is checked in advance; (3) Meeting IDs and passwords are sent directly to patients by e-mail. On the day of the examination, the patient visits the Support Department remotely to see the physician. Patient reception, Zoom setup and management, and payment are done by the Support Department. Figure 2 shows the numbers of online medical care patients at our hospital from April 2020 to April 2022. Although the number of patients was small at the beginning, as of April 2022, approximately 200 patients were receiving online medical care per month. In addition to online medical care, an online second opinion service was started in August 2020. About three or four online second opinions are provided per month.

## Fees for online medical care

Consultation fees for online medical care were in line with those for online medical care in normal times until April 2020, but thereafter provisional special measures were put in place and continued for 2 years (until 31 March 2022) (Table 1). No fee was set for the first visit in normal times, but in April 2020, under the provisional measure, a fee of 214 points was approved. Note that one point is worth 10 yen. The fee was increased to 251 points in April 2022. The fee for follow-up online consultations has remained more or less the same. In addition, initially, in the provisional measure period, the fee for online treatment of specific diseases was approximately 1.5 times that in normal times, but in April 2022 it was decreased to 87% of the face-to-face consultation fee for specific diseases, thus further increasing the number of patients eligible for online medical care. There are many types of fee for specific diseases. For example, the fee for outpatient guidance and management of intractable diseases is 270 points for face-to-face consultation, but decreases to 235 points for online medical care. Initially, in normal times, patients eligible for online medical care were limited to those whose physicians considered them eligible on the basis of the *Guidelines for the Appropriate Implementation of Online Medical Treatment*, including patients with intractable diseases or chronic headaches. However, in April 2020, patient requirements have been eased merely to those whose physicians considered them eligible on the basis of the Guidelines, and this expanded definition has continued since April 2022. However, prescribing restrictions are imposed on a patient's first visit. For physicians, e-learning was mandatory for online medical care in normal times, but during the provisional measure it was deferred. E-learning again became mandatory in April 2022.

**Table 1:** Fees for online medical care

		Normal times (until April 2020)	Provisional special measures (from April 2020 to end March 2022)	From April 2022
Medical fees	First visit	–	214 points	251 points
	Follow-up visit	71 points	74 points	73 points
	Medical treatment of specific diseases	100 points	147 points	87% of the face-to-face consultation fee
Patient requirements		Deemed eligible by physician on the basis of the *Guidelines for the Appropriate Implementation of Online Medical Treatment*, including patients with intractable diseases or chronic headache.	Deemed eligible on the basis of the revised Guidelines for the *Appropriate Implementation of Online Medical Treatment* (prescribing restrictions are imposed on a patient's first visit).	
E-learning requirements for physicians		Mandatory	Deferred	Mandatory

If the health care service concerned is covered by health insurance, patients must undergo face-to-face medical examination once every 3 months both in normal times and in provisional measures. One point is worth 10 yen.

Online medical care fees for the first visit are shown in Table 1. In addition to the fee for the first visit, a system utilization fee, determined at the discretion of each hospital, can be charged, and prescriptions can be made at the time of the first visit. However, narcotics, psychotropics, and high-risk drugs cannot be prescribed to patients whose underlying medical conditions are not known, and prescriptions for more than 8 days' supply cannot be made. Our hospital collects fees for both first and follow-up visits through a postpaid credit card service and a medication delivery service, thus reducing the burden on patients by not requiring them to come in.

## Advantages and disadvantages of online medical care

### Advantages of online medical care

The primary advantage of online medical care is that patients can receive medical care without coming to the hospital. This saves time and effort. For example, the time required to travel back and forth between the medical institution and the patient's home or office can be saved, especially if the patient is visiting from a remote location, and the cost of transportation to and from the hospital can be greatly reduced.

Second, it saves time spent waiting for payments. Our hospital has been able to save even more time by using a postpaid credit card service, a medication delivery service, and a system called “walk-through examinations”. Walk-through examinations can further reduce waiting time by allowing patients to be examined on a day different from the day of their consultation resulting in less waiting time than usual. Online medical care also makes it possible for patients to visit the hospital in between working hours and allows them to see the doctor in a place where their privacy can be protected.

Another advantage is that the patient can relax during the examination. In a hospital, patients may get nervous or excited in an unfamiliar place, but at home, they can relax when receiving treatment. In addition, there is no risk of infection at home.

Furthermore, the fees for online medical care are inexpensive, and patients have the advantage of paying less than they would in an actual face-to-face visit.

### Disadvantages of online medical care

Compared with face-to-face consultations, the first disadvantage of online medical care is that it is difficult to exchange information during the consultation. Visual examination and questioning are the primary focus of the consultation, and palpation and auscultation cannot be performed directly by the physician. However, nowadays, it may be possible for patients to communicate their physical information to physicians via various devices. Another disadvantage is that the patient must first visit a medical institution where online medical care is to be performed or a nearby medical institution for, for example, clinical examination, imaging, or blood collection. This means that if online medical care is to be performed while the physician is viewing the data, the examination results must be available in advance. Regardless, the patient must visit the relevant medical institution in person.

## Concluding remarks

As mentioned above, our hospital's online medical care has developed rapidly in response to the coronavirus outbreak, and at the time of writing (December 2022) it is being implemented stably. Patient satisfaction is high, and physicians have become accustomed to online medical consultations and are thus able to provide effective care. However, online medical care alone is not always sufficient, and it is important to provide face-to-face consultations on a regular basis whenever possible. Although online medical care will become increasingly useful with the development of information technology, combining it with regular face-to-face consultations is desirable so that emergency lesions and serious diseases not be overlooked.

## Funding

The author received no financial support for this research.

## Author contributions

RK read and approved the final manuscript.

## Conflicts of interest statement

The author declares that there are no conflicts of interest.
